# Luteal phase bleeding after IVF cycles: comparison between progesterone vaginal gel and intramuscular progesterone and correlation with pregnancy outcomes

**Published:** 2009-10-20

**Authors:** Sami Jabara, Kurt Barnhart, Joan C Schertz, Pasquale Patrizio

**Affiliations:** 1 Department of Obstetrics and Gynecology, Texas Tech University, Lubbock, Texas, USA; 2 Division of Reproductive Endocrinology and Infertility, Department of Obstetrics and Gynecology, University of Pennsylvania Medical Center, Philadelphia, Pennsylvania, USA; 3 EMD Serono, Inc., Rockland, Massachusetts, USA†; 4 Yale University Fertility Center, New Haven, Connecticut, USA

**Keywords:** Assisted reproductive technology, Progesterone, Luteal phase support, Progesterone vaginal gel

## Abstract

**Background:**

To compare luteal phase bleeding and pregnancy outcomes in normogonadotropic patients receiving progesterone vaginal gel (PVG) or intramuscular progesterone (IMP) injections.

**Methods:**

In this retrospective cohort study, data from 270 patients (292 cycles) undergoing day-3 fresh embryo transfer were analyzed. PVG, 90 mg daily (170 cycles) or IMP, 50 mg daily (122 cycles) began at egg retrieval.

**Results:**

Luteal phase bleeding was significantly more common in the PVG than the IMP group. No significant differences were observed in biochemical pregnancy or spontaneous abortion rates between the two groups. Patients who bled before the pregnancy test had significantly lower total and clinical pregnancy rates than non-bleeders. Total and ongoing pregnancy/delivery rates were higher in the PVG than IMP group, but did not achieve statistical significance.

**Conclusion:**

Luteal phase bleeding was more common in the PVG group than the IMP group, but pregnancy was successful in more patients in the PVG group. Luteal phase bleeding is prevented or delayed during IMP treatment, but patients who bled before the pregnancy test, whether using the gel or injected progesterone, had significantly reduced pregnancy rates compared with non-bleeders.

## Introduction

Progesterone has been traditionally used for luteal support in cycles of assisted reproduction. In addition to oral, intramuscular, and vaginal suppository formulations, a vaginal gel of micronized progesterone (Crinone^®^ 8%, Columbia Research Laboratories, Livingston, NJ) is available. This is micronized progesterone contained in a polycarbophil-based gel, which is designed to provide a controlled and sustained release of the hormone over a 48- to 72-hour interval after a single vaginal application [1,2].

Several randomized prospective trials and non-randomized prospective studies compared micronized progesterone vaginal gel (PVG) with intramuscular progesterone (IMP; progesterone in oil) in cycles of assisted reproductive technology (ART) [3–9]. Many of these studies noted no significant difference in clinical pregnancy rates, but reported a higher incidence of biochemical pregnancy rates and lower implantation rates with PVG compared with IMP. However, Abate et al. reported significantly higher ongoing pregnancy rates with IMP [4]. An interim analysis of a prospective, randomized trial, published in 2001, reported lower clinical pregnancy and live birth rates in PVG versus IMP ART cycles in which treatment with both progesterone products started 24 hours after oocyte retrieval [8]. Recently, the same group published interim outcomes from a subsequent prospective, randomized trial comparing both products; however, PVG was started 48 hours after oocyte retrieval while IMP treatment was started 24 hours after oocyte retrieval [9]. The rationale for the later start time with PVG was based on the product’s greater bioavailability versus IMP [1,10].

In our ART practice, PVG gained prompt acceptance with patients. However, in a number of cases, patients experienced luteal phase bleeding prior to the pregnancy test. Bleeding during the luteal phase of ART cycles and prior to the pregnancy test generates concern and distress for patients because they fear that the bleeding is a sign of treatment failure. Bleeding patterns were a measured outcome in several previous studies. Jobanputra et al. reported that only one of 44 patients using PVG bled before the pregnancy test but, nonetheless, delivered twins at term [5]. A greater proportion of PVG patients (27% IMP vs 42% PVG; P = 0.02) reported bleeding prior to the pregnancy test in the study by Yanushpolsky et al. but implantation and ongoing pregnancy rates were similar in both groups [9]. Roman et al. studied vaginal bleeding in women undergoing in vitro fertilization (IVF)/intracytoplasmic sperm injection (ICSI) and observed no shortened luteal phase when using vaginal micronized progesterone (200 mg three times a day [t.i.d.]) in soft gelatin capsules (Piette Pharmaceuticals, Brussels, Belgium) [11]. In 100 patients treated with IMP who did not become pregnant following ART, 67 reported bleeding within 17 days following oocyte retrieval [12].

Since there are few systematic studies addressing the issue of luteal phase bleeding with PVG or IMP as progesterone supplementation in ART cycles, we designed the current study with two objectives: (1) to compare the incidence and the onset of luteal phase bleeding after embryo transfer (ET) and prior to the serum pregnancy test; and (2) to compare the pregnancy outcomes between the two treatment groups and between patients who experienced luteal phase bleeding versus those who did not.

## Material and Methods

### Patient Population

Outcomes from 270 patients, with a total of 292 cycles, were analyzed. All patients were treated at the University of Pennsylvania Medical Center during 1999 and January through June 2000, were less than 40 years of age and underwent a fresh ET cycle. Patients undergoing egg donation cycles were excluded from this analysis. The PVG group (n = 159 patients, 170 cycles) received 90 mg daily (Crinone^®^ 8%) administered vaginally, while the IMP group (n = 111 patients, 122 cycles) received progesterone in oil, 50 mg daily, via intramuscular injection.

The protocol of ovarian stimulation was similar between groups, consisting of gonadotropin-releasing hormone agonist (GnRH-a) down-regulation with leuprolide acetate (0.5 mg, Lupron^®^, TAP Pharmaceuticals Products, Lake Forest, IL) commenced in the mid-luteal phase, followed by split doses of recombinant human follicle-stimulating hormone (r-hFSH; GONAL-f^®^, EMD Serono, Inc., Rockland, MA), and reduction of GnRH-a to 0.25 mg with the initiation of r-hFSH treatment. Ovarian stimulation was managed by two physicians only and adjusted depending on follicular response, as assessed by ultrasound monitoring and estradiol (E_2_) levels. When 3–4 follicles with a mean diameter of 20 mm or more were seen on ultrasound, human chorionic gonadotropin (hCG) (10,000 IU intramuscularly) was administered, followed by oocyte retrieval 36 hours later. ET was scheduled to occur 3 days following oocyte retrieval. For each patient, 2–4 embryos were transferred depending on the age of the patient and the quality of available embryos. In both groups, progesterone supplementation was initiated on the evening of egg retrieval.

### Analysis

This study was analyzed as a retrospective cohort study. Primary outcomes were the presence of early vaginal bleeding and pregnancy. An ongoing pregnancy was defined by the presence of a fetal heartbeat or a known, delivered infant after 20 weeks of gestation. For the purposes of this study, implantation rate was defined as the ratio of the number of gestational sacs on ultrasound divided by the total number of embryos transferred. Spontaneous abortion was defined as the loss of an ongoing pregnancy before 20 weeks of gestation. Biochemical pregnancy was defined as a positive β-hCG (> 5 mLU/mL) in a patient who failed to demonstrate a pregnancy by ultrasound. Luteal phase bleeding was defined as any bleeding or spotting reported to the office prior to the scheduled pregnancy test (day 14 after ET). This information was collected by the nursing staff and entered on a dated phone-consultation sheet.

Direct comparisons of selected variables between the two groups were performed using Student’s *t*-test, Chi-square test, or Fisher’s exact test where appropriate. All tests were two-sided and P < 0.05 was considered significant. Associations between outcomes and the progesterone treatment groups were assessed using both relative risks and odds ratios (ORs). As these measures gave similar results, the ORs are reported and were included in the subsequent multivariable logistic regression models.

To identify potential confounding variables and possible modifiers of the association between progesterone formulations and outcome (i.e. interactions between progesterone group and other variables), both unadjusted and stratified analyses were performed. Multivariable logistic regression was then used to estimate the OR for outcome (pregnancy) adjusted for all potential confounders. Variables were included in the logistic regression model if (a) they were biologically plausible, (b) they demonstrated P < 0.20 for the association with outcome in the unadjusted analyses, or (c) adjustment for the given variable changed the OR for PVG versus IMP by 10% [13,14]. The factors included in the final model were age (continuous variable) and etiology of infertility (categorical variable). Day-3 FSH serum concentration, starting dose of gonadotropin, number of oocytes retrieved, and the use of ICSI were not found to be confounders in the analysis and were not included in the final model.

## Results

Both groups were similar for day-3 FSH, etiology of infertility, gonadotropin dose, days of stimulation, number of follicles, E_2_ levels, number of eggs retrieved, use of ICSI, and endometrial stripe thickness on the day of hCG administration (Table 1).

The percentage of cycles with bleeding before the pregnancy test was significantly higher in the PVG group than the IMP group (37.1%, n = 63/170 vs 7.4% n = 9/122, P < 0.0001). Furthermore, for the patients who experienced luteal phase bleeding, the onset of bleeding occurred one day earlier in the PVG group (8.5 vs 9.5 days after ET), but this difference was not statistically significant (P = 0.09) (Table 2).

Total and ongoing pregnancy rates were higher in the PVG group than in the IMP group, although the difference did not reach statistical significance (52.3% vs 43.4%, P = 0.07 and 41.2% vs 33.6%, P = 0.08, respectively). The implantation rates, biochemical pregnancy rates, and spontaneous abortion rates were similar in both groups (24.0% vs 18.7%, P = 0.09; 14.6% vs 11.3%, P = 0.78, and 6.7% vs 5.7%, P = 0.77 in the PVG vs IMP groups, respectively).

Despite comparable E_2_ levels (3291 ± 208 pg/mL vs 3485 ± 121 pg/mL, P = 0.41), patients from both groups who bled prior to pregnancy testing had a significantly thicker endometrial stripe on the day of hCG administration (12.6 ± 2.9 mm vs 11.6 ± 2.3 mm, P = 0.004), when compared with the non-bleeders (Table 3). As a whole, cycles with luteal phase bleeding before the pregnancy test, whether using PVG or IMP, had significantly reduced total and clinical pregnancy rates compared with those without bleeding (19.4% vs 58.2%, P < 0.001 and 9.7% vs 47.3%, P < 0.001 respectively). The total pregnancy rate among cycles with luteal phase bleeding in the PVG group was 22.2% (14/63) while the ongoing pregnancy rate was 11.1% (7/63). None of the patients who bled early in the IMP group were pregnant (0/9) (Table 4).

## Discussion

Progesterone supplementation during the luteal phase has been considered standard treatment in most ART centers [15]. Despite various formulations and routes of administration available, the use of IMP is still the dominant choice in the United States. The major disadvantage of IMP is the discomfort associated with intramuscular injections, in addition to the potential risks for local inflammatory reactions and abscesses that could arise from repetitive injections of the oil vehicle [7]. The formulation of progesterone as a vaginal gel, from a practical perspective, is very convenient and well tolerated by patients with few side-effects. However, luteal phase bleeding prior to the scheduled pregnancy test generates significant anxiety. This occurrence coupled with questions of decreased pregnancy rates in cycles using this form of luteal progesterone supplementation prompted us to review our own extensive experience.

The data collected in the present study confirm the impression that patients receiving PVG for luteal support in fresh ET cycles were more likely to bleed prior to their pregnancy test than those treated with IMP. However, an interesting finding from the analysis is that neither pregnancy nor implantation rates significantly differed between the two treatment groups. In fact, our data show that most of the patients who experienced luteal phase bleeding (in both PVG and IMP groups) were either not pregnant or did not achieve an ongoing pregnancy. Thus, it seems that early bleeding was unrelated to implantation failure or pregnancy loss [16].

In some ART clinics, high E_2_ levels (>3200 pg/mL) observed on the day of hCG administration may indicate ovarian hyperstimulation. However, none of the patients with elevated E_2_ levels developed ovarian hyperstimulation syndrome in this study. Moreover, there was no significant difference between the IMP and PVG groups in terms of the mean E_2_ levels on the day of hCG administration (Table 1), nor was a significant difference observed in patients with and without luteal phase bleeding (Table 3). While the E_2_ levels observed in our program may be indicative of hyperstimulation in some ART clinics, the explanation may lie in variability of E_2_ assays between laboratories.

An intriguing, but certainly not surprising, finding of the present study was the observation that cycles with luteal phase bleeding had a significantly thicker endometrial stripe on the day of hCG administration compared with the non-bleeders (12.6 mm vs 11.6 mm, P = 0.004). Based on this finding, a subgroup of patients may benefit from increased progesterone supplementation based on the endometrial thickness measurement on the day of hCG administration. The data from the present study do not support either hypothesis, but point to the need for additional investigations.

First-trimester bleeding in spontaneous pregnancies is not uncommon, occurring about 20% of the time [17]. Following ART, the reported incidence of first-trimester bleeding is higher, ranging from 29% to 36% [18,19]. As noted previously, luteal phase bleeding was not a measured outcome in the majority of previous comparative studies between PVG and IMP. Roman et al. addressed the issue of bleeding during the luteal phase of IVF/ICSI cycles, yet the authors reported no shortened luteal phase [11]. In that study, however, vaginal gelatin capsules of micronized progesterone (at a dose of 200 mg t.i.d.) instead of PVG were utilized, and there was no IMP control group. Yanushpolsky et al. reported the results of an interim analysis from a prospective, randomized study comparing the efficacy and tolerability of PVG and IMP for luteal phase support in ART [9]. Although a significantly higher proportion of patients receiving PVG than IMG reported bleeding before the first pregnancy test (42% vs 27%, respectively; P = 0.02), similar implantation, pregnancy, and spontaneous abortion rates were found in the two treatment groups.

Of greater concern to patients and medical staff alike has been the suggestion that luteal phase support with PVG may compromise implantation and pregnancy rates. Conflicting results have been reported in the literature regarding this issue. Several prospective studies have demonstrated that PVG is as effective as IMP for luteal phase supplementation in ART cycles [3,5–7]. However, one randomized, controlled (open label) study noted that PVG resulted in significantly lower embryo implantation, clinical pregnancy, and live birth rates compared with the pregnancy outcomes in women supplemented with IMP [8]. Nonetheless, this study might have been limited by type I error and bias despite its randomized design [20], and a subsequent study from the same group showed no differences in treatment outcomes [9]. Others have reported significantly higher implantation and clinical pregnancy rates with PVG compared with IMP in ICSI but not classic IVF cycles. These observations suggest that the differences may be attributed to a direct effect of progesterone on the oocyte, which is deprived of progesterone due to decoronization prior to sperm injection. Because progesterone levels are significantly higher in the local environment after PVG administration, a possible ‘rescue effect’ of progesterone on both the early embryo and endometrium may occur in ICSI cycles [21].

Several limitations may be noted regarding this study. For the purpose of our analysis, the number of embryos transferred and multiple pregnancy rates were inadvertently excluded when data were extracted from the patients’ charts. However, the overall data from our center were published in the *Assisted Reproductive Technology Success Rates National Summary and Fertility Clinic Reports* for the time periods during which these patients were treated [22,23]. The mean number of fresh embryos transferred ranged from 2.5 for patients aged under 35 years to 3.3 for those aged 38–40 years. The percentage of twin pregnancies ranged from 25% for patients aged under 35 years to 40% for those aged 35–37 years. The percentage of triplet pregnancies ranged from 0% for patients aged under 35 years to 15.4% for those aged 35–37 years.

An important observation relevant to clinical practice is confirmation of the effectiveness of PVG for luteal support and the observation that, contrary to other reports, biochemical pregnancy rates were similar to patients in the IMP group [5,6]. In addition, it should be noted that a trend towards higher implantation and pregnancy rates was also observed in the PVG users (Table 2). However, this observation may be explained by the fact that patients in the PVG group were significantly younger than those in the IMP group.

In conclusion, the results of this study suggest that patients using PVG should be informed that they might experience vaginal spotting prior to their pregnancy test, but they should be reassured that this bleeding does not indicate treatment failure nor does it affect their chances of achieving a pregnancy. They should be counseled that even if they bleed early on, they should continue taking PVG until the pregnancy test (12–14 days after the ET). A prospective, randomized trial of sufficient statistical power is needed to confirm these data and to further evaluate the relationship between endometrial stripe thickness and early vaginal bleeding.

## Figures and Tables

**Table 1.** Clinical characteristics in ART cycles treated with progesterone vaginal gel (PVG) versus intramuscular progesterone (IMP)

Variable	PVG group	IMP group	P value
Number of patients (cycles)	159 (170)	111 (122)	
Mean ± SD age, years	33.1 ± 4.7	34.4 ± 3.5	0.004*
Mean ± SD day-3 FSH, mLU/mL	7.0 ± 2.1	7.0 ± 2.1	0.87*
*Etiology of infertility*			0.16†
Unexplained, %	23.5	19.7	
Endometriosis, %	17.7	18.8	
Tubal, %	21.7	33.6	
Male, %	29.4	17.2	
PCO/anovulation, %	4.1	4.9	
Pelvic adhesions, %	1.1	0.8	
Uterine, %	0.6	0.8	
Other, %	1.8	4.1	
Mean ± SD number of follicles	19.7 ± 7.5	18.8 ± 11.9	0.50*
Mean ± SD number of eggs retrieved	13.8 ± 5.6	11.5 ± 5.8	0.08*
ICSI, %	21.8	16.3	0.25†
Mean ± SD E_2_ level on day of hCG administration, pg/mL	3577 ± 1453	3316 ± 1565	0.14*
Mean ± SD ES thickness on day of hCG administration, mm	11.5 ± 2.3	12.1 ± 2.6	0.06*
Fertilization rate, %	54	55	0.85†

E_2_ = estradiol; ES = endometrial stripe; FSH = follicle-stimulating hormone; hCG = human chorionic gonadotropin; ICSI = intracytoplasmic sperm injection; PCO = polycystic ovary/ovaries; SD = standard deviation.

* Student’s *t*-test;

^†^ Chi-square test.

**Table 2.** Luteal phase bleeding and pregnancy outcomes in ART cycles treated with progesterone vaginal gel (PVG) versus intramuscular progesterone (IMP)

	PVG group	IMP group	Odds ratio (crude)	P*-value* (crude)	Odds ratio (adjusted)	P value (adjusted)
Number of patients (cycles)	159 (170)	111 (122)	–	–	–	–
Bleeding before day 14	63/170 (37.1%)	9/122 (7.4%)	6.59 (3.2–13.52)	< 0.001*	6.25 (3.0–13.01)	< 0.0001*
Mean day of bleeding after hCG	8.5	9.5	–		–	0.09†
Implantation rates	24.0%	18.7%	1.80 (0.81–3.99)	0.15	2.09 (0.89–4.91)	0.09*
Total pregnancies	89/170 (52.3%)	53/122 (43.4%)	1.43 (0.9–2.28)	0.13	1.58 (0.96–2.61)	0.07*
Ongoing pregnancies	70/170 (41.2%)	41‡/122 (33.6%)	1.38 (0.85–2.24)	0.19	1.60 (0.95–2.69)	0.08*
Biochemical pregnancies	13/89 (14.6%)	6/53 (11.3%)	1.34 (0.48–3.77)	0.58	1.17 (0.39–3.52)	0.78*
Spontaneous abortions	6/89 (6.7%)	3/53 (5.7%)	1.20 (0.29–5.03)	0.80	1.25 (0.27–5.71)	0.77*

* Chi-square test;

^†^ Student’s *t*-test;

^‡^ Three patients with positive pregnancy tests were subsequently lost to follow-up.

**Table 3.** Endometrial stripe thickness (ES) and estradiol levels (E_2_) in ART cycles with luteal phase bleeding versus non-bleeding

	Luteal phase bleeding	Non-bleeding	P value
Number of cycles	72	220	–
Mean ± SD E_2_ on day of hCG administration, pg/ml	3291 ± 208	3485 ± 121	0.41*
Mean ± SD ES thickness on day of hCG administration, mm	12.6 ± 2.9	11.6 ± 2.3	0.004*
Total pregnancies (%)	14/72 (19.4)	128/220 (58.2)	0.001†
Ongoing pregnancies (%)	7/72 (9.7)	104/220 (47.3)	< 0.001†

SD = standard deviation; hCG = human chorionic gonadotropin.

* Student’s *t*-test;

^†^ Chi-square test.

**Table 4.** Pregnancy rates* in cycles with luteal phase bleeding using progesterone vaginal gel (PVG) or intramuscular progesterone (IMP)

	n	Total pregnancy rate*	Ongoing pregnancy rate*
All patients with luteal phase bleeding, n (%)	72	14 (19.4)	7 (9.7)
PVG group, n (%)	63	14 (22.2)	7 (11.1)
IMP group, n (%)	9	0 (0)	0 (0)

* Number of pregnancies and percentage of total pregnancies.

## References

[1] Miles RA Paulson RJ Lobo RA Press MF Dahmoush L Sauer MV Pharmacokinetics and endometrial tissue levels of progesterone after administration by intramuscular and vaginal routes: a comparative study Fertil Steril 1994 62 485 490 8062942 10.1016/s0015-0282(16)56935-0

[2] Fanchin R De Ziegler D Bergeron C Righini C Torrisi C Frydman R Transvaginal administration of progesterone Obstet Gynecol 1997 90 396 401 9277651 10.1016/s0029-7844(97)00270-6

[3] Gibbons WE Toner JP Hamacher P Kolm P Experience with a novel vaginal progesterone preparation in a donor oocyte program Fertil Steril 1998 69 96 101 9457941 10.1016/s0015-0282(97)00457-3

[4] Abate A Perino M Abate FG Brigandì A Costabile L Manti F Intramuscular versus vaginal administration of progesterone for luteal phase support after in vitro fertilization and embryo transfer. A comparative randomized study Clin Exp Obstet Gynecol 1999 26 203 206 10668157

[5] Jobanputra K Toner J Denoncourt R Gibbons WE Crinone 8% (90 mg) given once daily for progesterone replacement therapy in donor egg cycles. Fertil Steril 1999 72 980 984 10593367 10.1016/s0015-0282(99)00390-8

[6] Chantilis S Zeitoun K Patel S Johns DA Madziar VA McIntire DD Use of Crinone vaginal progesterone gel for luteal support in in-vitro fertilization cycles Fertil Steril 1999 72 823 829 10560985 10.1016/s0015-0282(99)00362-3

[7] Schoolcraft WB Hesla MJ Gee M Experience with progesterone gel for luteal support on a highly successful IVF programme Hum Reprod 2000 15 1284 1288 10831556 10.1093/humrep/15.6.1284

[8] Propst AM Hill JA Ginsburg ES Hurwitz S Politch J Yanushpolsky EH A randomized study comparing Crinone 8% and intramuscular progesterone supplementation in in vitro fertilization-embryo transfer cycles Fertil Steril 2001 76 1144 1149 11730742 10.1016/s0015-0282(01)02872-2

[9] Yanushpolsky E Hurwitz S Greenberg L Racowsky C Hornstein MD Comparison of Crinone 8% intravaginal gel and intramuscular progesterone supplementation for in vitro fertilization/embryo transfer in women under age 40: interim analysis of a prospective randomized trial Fertil Steril 2008 89 485 487 17572412 10.1016/j.fertnstert.2007.03.006

[10] Cicinelli E de Ziegler D Bulletti C Matteo MG Schonauer LM Galantino P Direct transport of progesterone from vagina to uterus Obstet Gynecol 2000 95 403 406 10711552 10.1016/s0029-7844(99)00542-6

[11] Roman E Aytoz A Smitz JE Faguer B Camus M Van Steirteghem AC Devroey P Analysis of the bleeding pattern in assisted reproduction cycles with luteal supplementation using vaginal micronized progesterone Hum Reprod 2000 15 1435 1439 10875847 10.1093/humrep/15.7.1435

[12] Gürbüz B Yalti S Fiçicioglu C Delikara N Alpay Z Bleeding patterns in women using intramuscular progesterone for luteal support in in-vitro fertilisation cycles J Obstet Gynaecol 2003 23 267 270 12850858 10.1080/01443610310000100079

[13] Mickey RM Greenland S The impact of confounder selection criteria on effect estimation Am J Epidemiol 1989 129 125 137 2910056 10.1093/oxfordjournals.aje.a115101

[14] Maldonado G Greenland S Simulation study of confounder selection strategies Am J Epidemiol 1993 138 923 936 8256780 10.1093/oxfordjournals.aje.a116813

[15] Soliman S Daya S Collins J Hughes EG The role of luteal phase support in infertility treatment: a meta-analysis of randomized trials Fertil Steril 1994 61 1068 1076 8194619 10.1016/s0015-0282(16)56758-2

[16] Penzias AS Alper MA Luteal support with vaginal micronized progesterone gel in assisted reproduction Reprod Biomed Online 2003 6 287 295 12735861 10.1016/s1472-6483(10)61847-0

[17] Everett C Incidence and outcome of bleeding before the 20th week of pregnancy: prospective study from general practice BMJ 1997 315 32 34 9233324 10.1136/bmj.315.7099.32 PMC2127042

[18] Goldman JA Ashkenazi J Ben-David M Feldberg D Dicker D Voliovitz I First trimester bleeding in clinical IVF pregnancies Hum Reprod 1988 3 807 809 3220946 10.1093/oxfordjournals.humrep.a136787

[19] Hofmann GE Gundrum CL Drake LM Bertsche AB Frequency and effect of vaginal bleeding on pregnancy outcome during the first 3 weeks after positive beta-hCG test results following IVF-ET Fertil Steril 2000 74 609 613 10973668 10.1016/s0015-0282(00)00682-8

[20] Barnhart K Sammel M The truth is out there: a guide to the pitfalls of interpreting evidence-based medicine in studies of human reproduction Fertil Steril 2002 77 223 228 11821075 10.1016/s0015-0282(01)03207-1

[21] Manno M Marchesan E Cicutto D Zadro D Favretti C Tomei F Greater implantation and pregnancy rates with vaginal progesterone in intracytoplasmic sperm injection but not in in vitro fertilization cycles: a retrospective study Fertil Steril 2005 83 1391 1396 15866574 10.1016/j.fertnstert.2004.11.054

[22] Centers for Disease Control and Prevention Assisted reproductive technology success rates Available from: http://www.cdc.gov/ART/ArchivedARTPDFs/1999ART_accessible_erat.pdf

[23] Centers for Disease Control and Prevention Assisted reproductive technology success rates Available from: http://www.cdc.gov/ART/ArchivedARTPDFs/ART2000.pdf

